# Comparison of multi-tissue aging between human and mouse

**DOI:** 10.1038/s41598-019-42485-3

**Published:** 2019-04-17

**Authors:** Jujuan Zhuang, Lijun Zhang, Shuang Dai, Lingyu Cui, Cheng Guo, Laura Sloofman, Jialiang Yang

**Affiliations:** 1 School of Science, Dalian Maritime University, Dalian, Liaoning 116026 P. R. China; 2 Center for Infection and immunity, Columbia University, New York City, New York, USA; 3 Department of Genetics and Genomic Sciences, Icahn School of Medicine at Mount Sinai, New York City, New York, USA; 4 Geneis (Beijing) Co. Ltd, Beijing, 100102 P. R. China

**Keywords:** Computational biology and bioinformatics, Gene expression, Gene expression, Computational biology and bioinformatics, Gene expression

## Abstract

With the rapid growth of the aging population, exploring the biological basis of aging and related molecular mechanisms has become an important topic in modern scientific research. Aging can cause multiple organ function attenuations, leading to the occurrence and development of various age-related metabolic, nervous system, and cardiovascular diseases. In addition, aging is closely related to the occurrence and development of tumors. Although a number of studies have used various mouse models to study aging, further research is needed to associate mouse and human aging at the molecular level. In this paper, we systematically assessed the relationship between human and mouse aging by comparing multi-tissue age-related gene expression sets. We compared 18 human and mouse tissues, and found 9 significantly correlated tissue pairs. Functional analysis also revealed some terms related to aging in human and mouse. And we performed a crosswise comparison of homologous age-related genes with 18 tissues in human and mouse respectively, and found that human Brain_Cortex was significantly correlated with Brain_Hippocampus, which was also found in mouse. In addition, we focused on comparing four brain-related tissues in human and mouse, and found a gene–*GFAP*–related to aging in both human and mouse.

## Introduction

Aging population is a huge challenge faced by all countries around the world. Given the rapid growth of the global aging population, researchers are interested in identifying treatments that would delay the physiological, metabolic, and functional decline that gradually occurs in various systems, organs, and tissues of the body as they age. Additionally, it is well known that aging is closely related to a variety of complex diseases including partial cancer, Alzheimer’s disease, Parkinson’s disease, type 2 diabetes, multiple cardiovascular diseases, and neurodegenerative diseases etc.^1–6^. While understanding the biological basis of the aging process is a major scientific challenge that will require integration of molecular, cellular, genetic and physiological approaches^7^. We hope that we can use model organisms instead of humans to do some research on diseases and drugs, and ultimately achieve the purpose of delaying aging and reducing the occurrence of diseases related to aging. However, it is not clear whether the aging research done on mice is effective on humans. Therefore, in this paper we compared the aging mechanism of human and mouse on multiple tissues at the level of gene expression.

With the advent of various high-throughput sequencing technologies, such as RNA-seq^8^, the development and improvement of the novel gene expression databases has made it possible to define aging processes by analyzing the transcriptional differences between the young and old. The Genotype-Tissue Expression (GTEx) Portal (https://www.gtexportal.org/home/)^9,10^ is a resource database generated from an analysis of RNA sequencing data from 1641 samples across 43 tissues from 175 individuals whose ages range from 20 to 79. This provides a data basis for us to study the relationship between gene expression and aging in human tissues.To elucidate the aging differences between humanand mouse at the molecular level, we systematically assessed the relationship between human and mouse aging by comparing age-related gene expression. We hope that this study, along with future work, will help researchers to justify the utilization of model organisms in research on aging and aging related diseases.

An early study of aging between species was performed by McCarroll *et al*. comparing transcriptional changes among C. elegans, D. melanogaster, Saccharomyces cerevisiae and Homosapiens, and showed that most of the changes associated with aging are species-specific, aging between C. elegans and D. melanogaster is highly conserved^11^. Khaitovich *et al*. analyzed gene expression in various brain regions of human and chimpanzees, and clarified that human and chimpanzee have significant differences in aging^12^. Zahn *et al*. provided the AGEMAP gene expression database and explored similar age-regulated genes and gene sets in different species: M. musculus, H. sapiens, D. melanogaster, and C. elegans, and it was eventually found that there was no overall correlation between mouse and human aging- related expression changes, similarity was only found in several specific gene sets^13^. And Yang *et al*. also showed that the aging genes were significantly different between human and mouse^14^. The results were not ideal since the datasets they used with small size of samples or with poor data quality.

**Figure 1:**
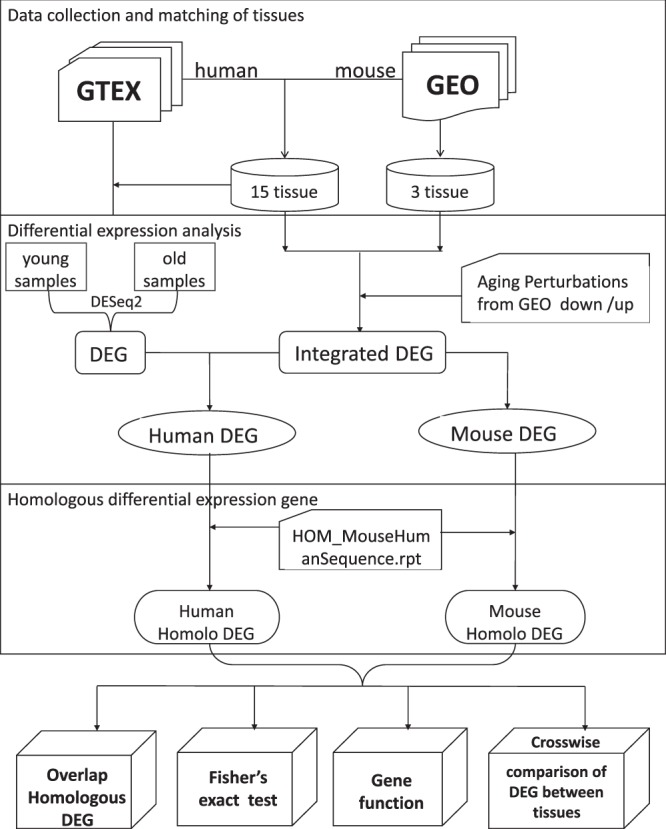
Workflow of the aging project.

In this work, we studied age-related genes in 18 tissues of human and mouse (see Fig. 1). We applied Deseq2 to perform differential expression analysis on the young and the old samples of 15 human tissues collected from GTEx database, and compared the results with DEGs of 15 mouse tissues studied by Wang *et al*. with CD algorithm^15^ from Gene Expression Omnibus(GEO) data^16^ (see Table 1), we also compared the DEGs of 3 pairs of human and mouse tissues studied by Wang *et al*. from GEO data^15^ (see Table 2). Then we performed functional analysis on these DEGs. Furthermore, we compared the aging DEGs in 18 tissues crosswise for human and mouse respectively, especially contrasted the four tissues associated with the brain. Since human and mouse DEGs are obtained by different algorithms, we applied CD and DESeq2 to analyze the DEGs of human Adipose_Subcutaneous respectively in order to compare the two algorithms.

**Table 1:** The 15 tissues of human and mouse from GTEx database and GEO database respectively.

Human (GTEx)	Mouse(GEO)
Adipose_Subcutaneous	peripheral adipocyte
Adipose_Visceral	bone marrow adipocyte
Artery_Aorta	thoracicaorta
Brain_Cerebellum	cerebellum
Brain_Cortex	neocortex
Brain_Hippocampus	hippocampus
Heart_Atrial	heart
Heart_Left_Ventricle	cardiac ventricle
Kidney_Cortex	kidney
Liver	liver
Lung	Lung
Muscle_Skeletal	skeletal muscle
Ovary	ovary
Spleen	spleen
Small_Intestine_Terminal_Ileum	Small_Intestine_Terminal_Ileum

**Table 2:** The 3 tissues of human and mouse from GEO database.

Human (GEO)	Mouse (GEO)
brain	Brain (frontal cortex)
hematopoietic stem cells	hematopoietic stem cells
retinal	retinal

## Results

### DEGs between young and old human samples from GTEx

GTEx Portal is a resource database generated from an analysis of RNA sequencing data of 1641 samples across 43 tissues from 175 individuals, built to help researchers study the relationship between genetic variation and gene expression in human tissues. In this paper, we used 15 human tissues RNA-seq datasets in GTEx for differential analysis (see Table 1). There are many methods for differential expression analysis of RNA-Seq data so far^17–23^. Anders *et al*. have proved that DESeq is the most conservative method among edgeR, DESeq, ShrinkSeq, NBPSeq, TSPM, voom + limma, vst + limma, baySeq, EBSeq and SAMseq^20^. But Love *et al*. clarified that DESeq2 is better than DESeq^24^. So, in this paper, for 15 human tissues from GTEx^9^, we used edgeR^25,26^, DESeq^27^ and DESeq2^24^ to call differential genes in the young and old samples, and we call these DEGs as “age-related genes”. We found that DESeq2 is more sensitive than the other two methods and the number of age-related genes obtained by DESeq2 is the largest.

We summarized the number of age-related genes in 15 human tissues in Table 3. And these genes can be found in the Supplementary Dataset 1.

**Table 3:** Overview of differential expression analysis in 15 human and mouse tissues.

GTEX(human)						GEO(mouse)			Overlap Homolo DEGs	Fisher’s exact test	
Tissues	Sample size			DEseq2 DEGs	Homolo DEGs	Tissues	CD DEGs	Homolo DEGs		p-value	adjusted p-value
	young	old	overall								
Adipose_Subcutaneous	36	52	88	4976	3707	peripheral adipocyte	1128	992	232	0.001176499	1.294149e-02
Adipose_Visceral	23	35	58	6101	4123	bonemarrow adipocytes	1289	1120	263	0.05009622	4.007698e-01
Artery_Aorta	34	33	67	6557	4410	thoracicaorta	1024	912	273	0.000135972	1.631664e-03
Brain_Cerebellum	7	28	35	1569	1259	cerebellum	918	675	53	0.1833056	1.000000e + 0
Brain_Cortex	5	25	30	2912	2292	neocortex	1314	1191	225	1.387631e-08	1.942683e-07
Brain_Hippocampus	5	26	31	3109	2392	hippocampus	6222	5215	820	2.203335e-13	3.525336e-12
Heart_Atrial	16	33	49	267	199	heart	1743	1559	22	0.1757766	1.000000e + 0
Heart_Left_Ventricle	28	28	56	2694	1109	cardiac ventricle	1157	1024	152	0.003899278	3.899278e-02
Kidney_Cortex	5	7	12	1	1	kidney	1775	1572	0	1	1.000000e + 0
Liver	9	19	28	130	108	liver	5512	4756	48	7.773007e-06	1.010491e-04
Lung	36	48	84	8785	6078	lung	1904	1715	503	0.9896731	1.000000e + 0
Muscle_Skeletal	58	67	125	6329	4586	skeletal muscle	1045	953	249	0.2691384	1.000000e + 0
Ovary	15	18	33	1180	890	Ovary	787	726	33	0.7785577	1.000000e + 0
Small_Intestine	17	8	25	14	11	Small_Intestine	973	816	0	1	1.000000e + 0
Spleen	17	7	24	104	39	Spleen	600	493	4	0.04076562	3.668906e-01

### DEGs between the young and the old samples from GEO data

GEO^28–30^ is a database provided by the National Center for Biotechnology Information (NCBI). In the study, in order to compare gene expression differences in young and old mice, we downloaded age-related genes expression profiles of multiple tissues in mouse from the GEO database^29^. Since these data are microarray data^31^, we used limma algorithm to perform differential expression analysis. However, the numbers of age-related genes were smaller than those derived by Wang *et al*.^15^, so we directly used the results of the DEGs they obtained. For the corresponding **GSE** (**Series**) information of each tissue, see Supplementary Table S1 and Supplementary Dataset 2 shows the detailed summary of DEGs in 15 mouse tissues (matching the tissues obtained from GTEx) from GEO database obtained by Wang Z *et al*.

Moreover, we also summarized the age-related genes of three pairs of human and mouse tissues (see Table 2) that are matched exactly from GEO database. Table 4 provides the numbers of age-related genes integrated in brain, retinal_periphery and hematopoietic_ stem_cell of human and mouse respectively. For a more detailed summary of age-related genes, see Supplementary Dataset 2.

**Table 4:** Overview of differential expression analysis in 3 human and mouse tissues from GEO database

GEO (human)			GEO(mouse)			Overlap HomoloDEG	Fisher’s exact test	
Tissues	CD DEG	Homolo DEG	Tissues	CD DEG	Homolo DEG		pvalue	p.adjust
brain	1836	1589	**brain**	2274	2020	350	2.73741e-46	4.653597e-45
retinal	600	549	**retinal**	3603	3221	328	9.536745e-112	1.716614e-110
hematopoietic_stem_cells	1214	943	**hematopoietic_stem_cells**	600	502	61	3.168386e-13	4.752579e-12

### Comparison of human and mouse homologous age-related genes

To compare gene expression across mouse and human fairly, we restricted our genes in both species to homologous genes, or genes that are at least 80% similar in both species. Most homologous genes have the same or similar biological functions, and the regulatory pathways are similar. Homologous genes were selected using HOM_MouseHuman Sequence.rpt from MGI Data and Statistical Reports (http://www.informatics.jax.org/downloads/reports/index.html). More detailed information on these homologous genes can be found in Supplementary Dataset 3.

In column 6 of Table 3 and column 3 of Table 4, we showed homologous age-related genes in 18 human tissues, the numbers of which range from 1 to 6078. The numbers of homologous age-related genes in 18 mouse tissues range from 493 to 5215, as shown in column 9 of Table 3 and column 6 of Table 4.

The comparative analysis of human and mouse homologous age-related genes was mainly carried out from three perspectives:

#### Quantifying the overlap of human and mouse homologous age-related genes

The overlap of homologous age-related genes of 18 human and mouse tissues can be seen in column 10 of Table 3 and column 7 of Table 4 respectively, and the numbers of which range from 0 to 820. In kidney and small intestine, there aren’t overlapping homologous age-related genes between human and mouse.

#### The Fisher’s exact test

To get a statistically demonstration, we performed fisher’s exact test on homologous age-related genes of human and mouse 18 tissues. For example, in terms of human Liver and mouse liver, we used the total homologous genes of human and mouse as the background (14212), and made fisher’s exact test on aging genes of human Liver (108) and aging genes of mouse liver (4756) (Table S2). In Tables 3 and 4, we show the p-values of 18 pairs of tissues obtained by fisher’s exact test, and their adjusted p-values. We define tissues with adjusted p-value < 0.05 as tissues that are significantly correlated in human and mouse. There are 9 pairs of tissues that are significantly correlated, and the three pairs of tissues from GEO database are more similar. Also, we note that the three pairs of tissues data are all microarray data, and the same algorithm was used to analyze the DEGs.

#### Enriched functions of homologous age-related genes

In this section, we performed gene functional analysis with David^32^ on homologous age-related genes obtained from 18 pairs of human and mouse tissues, and adjusted enrichment p-values using a Benjamini-Hochberg procedure. Corrected p-values were considered significant if p_Ben_ < 0.05. We showed the top 10 enriched terms for every pair of tissues of human and mouse in Table S3, and detailed results can be found in Supplementary Dataset 4. The number of overlapping GO and KEGG terms^33,34^ in 18 human and mouse tissues ranges from 0 to 68 (see Table S4).

As shown in Table S3, the functional enrichment analysis revealed that homologous aging-related genes were significantly enriched in GO:0031012~extra cellular matrix between human Heart_Atrial_Appendage and mouse heart. And DR Sell *et al*. have proved that the extra cellular matrix undergoes progressive changes during senescence^35^. We also see that GO:0005615~extracellular space is enriched between human spleen and mouse spleen.

We also found that Phosphoprotein was the term of the homologous age-related genes enriched in Ovary, Brain_Cerebellum, Adipose_Visceral_ (Omentum), Lung, Heart_Left_Ventricle, Artery_Aorta, Muscle_Skeletal, Brain_Cortex, Brain_Hippocampus, brain and Adipose_ Subcutaneous significantly between human and mouse. And Kahn A *et al*. have declared that changes in cellular expression of phosphoprotein are linked to insulin resistance, tumor cell invasion, and cellular senescence^36,37^. And homologous age-related genes relating to the cytoplasm were significantly enriched in Ovary, Adipose_Visceral_(Omentum), Lung, Heart_ Left_Ventricle, Artery_Aorta, Muscle_Skeletal, Brain_Cortex and Brain_Hippocampus between human and mouse. Dou Z *et al*. have discovered that the cytoplasmic chromatin-cGAS -STING pathway promotes the senescence-associated secretory phenotype in primary human cells and in mouse^38^.

### Crosswise comparison of homologous age-related genes between tissues

Here, we carried out pair wise comparison of homologous age-related genes of 18 tissues in human and in mouse separately. A more detailed summary of overlapping genes and fisher’s exact test p-values can be found in Supplementary Dataset 5.

When analyzing human homologous age-related genes, for Adipose_Visceralis, as an example, the tissue with the biggest overlap of homologous age-related genes is lung. Inomata *et al*. have found an association between the visceral adipose tissue level and lung function^39^. And excessive abdominal visceral fat contributes to increase plasma IL-6, which, in turn, is strongly associated with all-caused and cause-specific mortality in older persons with obstructive lung disease^40,41^. We also found that in the comparison of human 18 tissues, the two tissues with the highest number of overlapping DEGs are Muscle_Skeletal and Lung. This is consistent with the findings of Serres *et al*. who found that impaired skeletal muscle endurance in patients with chronic obstructive pulmonary disease was associated with altered lung function and reduction in associated physical activity^42^. Furthermore, the p-value obtained by fisher’s exact test indicates that the tissue most correlated with Adipose_Subcutaneous is Muscle_Skeletal (2.793932e-55), and Brain_Cortex is significantly correlated with Brain_Hippocampus (8.349845e-220).

In terms of 18 mouse tissues, for neocortex, the tissue with the biggest number of overlapping homologous age-related genes is Hippocampus, the overlapping number is 849 and the p-value of fisher’s exact test is 1.169441e-199. This result is consistent with human.

### Comparison of homologous age-related genes in human Brain_Cerebellum, Brain_Cortex, Brain_Hippocampus and brain (from GEO)

**Figure 2:**
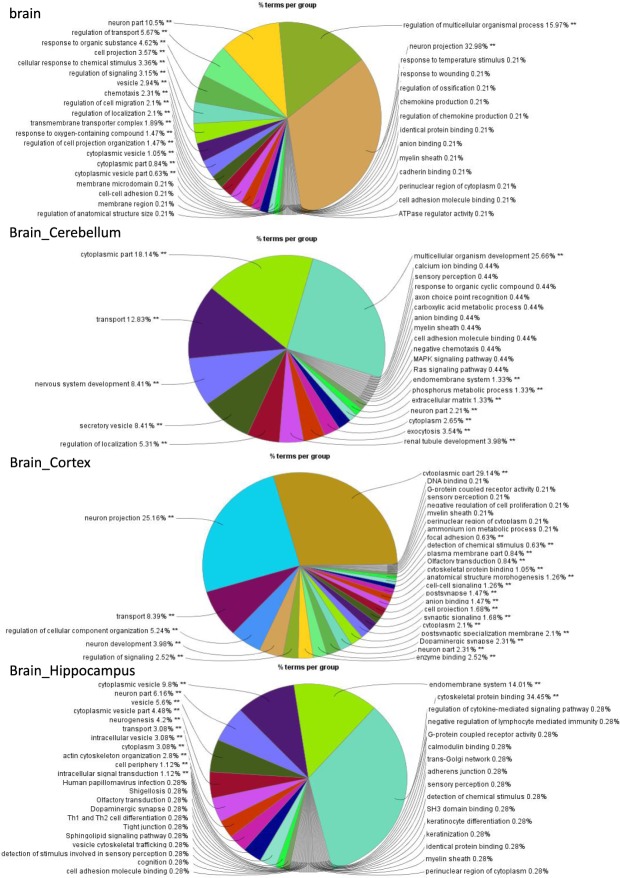
Functional groups in ClueGO Overview. ClueGO analysis of DEGs in Brain_Cerebellum, Brain_Cortex, Brain_Hippocampus and brain from human donors. Overview chart with functional groups including specific terms for DEGs. The percentage of genes per term is shown in each group.

Here, we did a more in-depth study of the four tissues associated with human brain: Brain_Cerebellum, Brain_Cortex, Brain_Hippocampus and brain (from GEO). 39 homologous age-related genes are overlapped in these four tissues (see Table S5). Biological interpretation of these DEGs was carried out using ClueGO v2.5.1^43^ in Cytoscape^44^, we reserved the terms with p-value < 0.05 (see Fig. 2), and got 56 overlapping terms (see Table S6).

### Comparison of homologous age-related genes in mouse cerebellum, neocortex, hippocampus and brain

**Figure 3:**
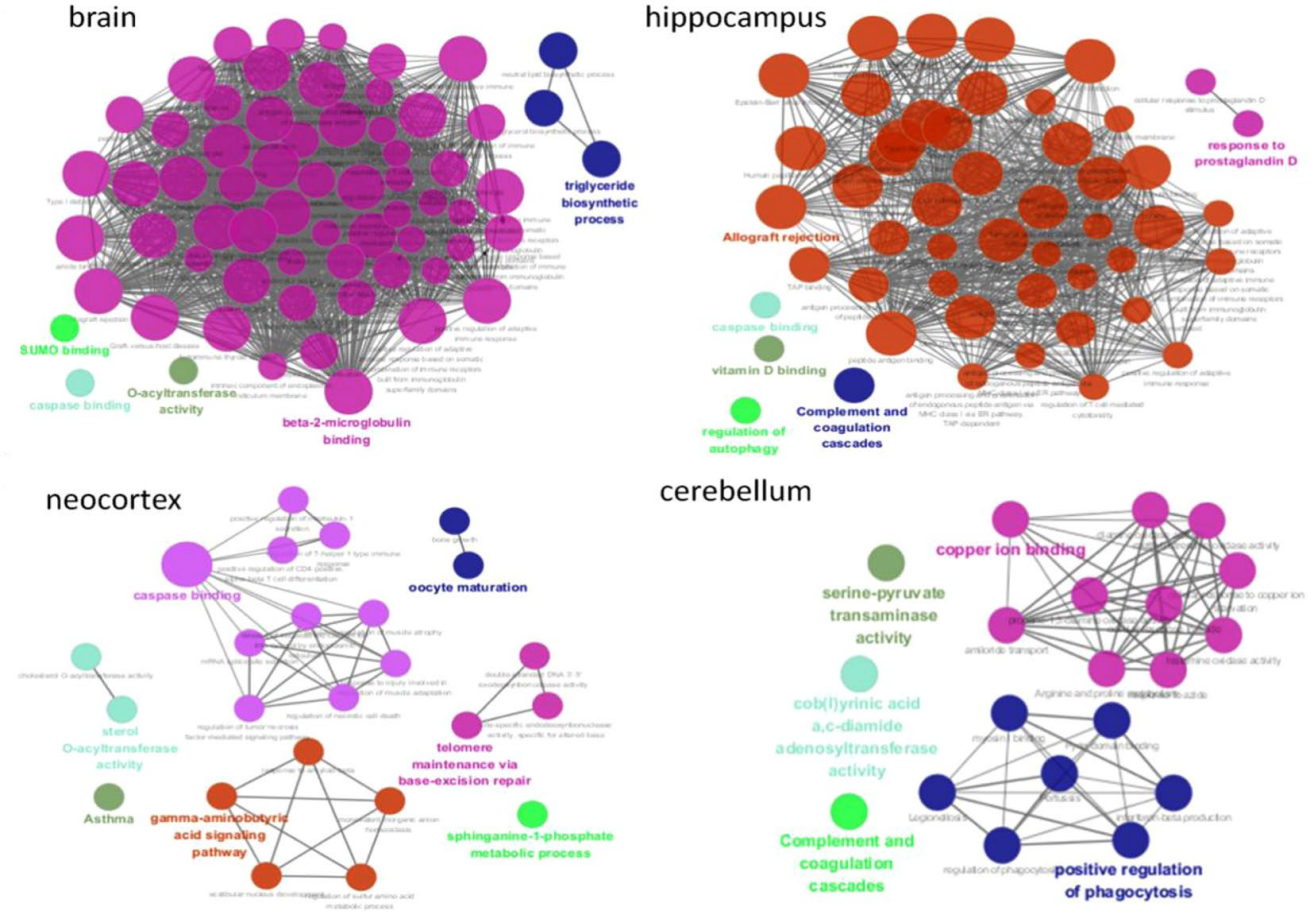
Functionally grouped networks on cerebellum, neocortex, hippocampus and brain for mouse. Functionally grouped network with terms as nodes linked based on their kappa score level (≥ 0.4), where only the label of the most significant term per group is shown. Each node in the figure represents a term, and the node size represents the term enrichment significance. Functionally related groups partially overlap. The connection between the nodes reflects the correlation between the terms, and the color of the node reflects the enrichment classification of the node.

Similarly, we made a further comparison of the four tissues associated with mouse brain: the cerebellum, neocortex, hippocampus and brain. There are just 8 overlapping age-related DEGs among these four tissues (see Table S5). As the studying process of human brain, the results of mouse brain biological interpretation are in Fig. 3, and there is no overlapping terms among these four tissues in mouse.

It is worth noting that GFAP appears in both human and mouse overlapping DEGs list. Middeldorp *et al*. have already proved that the astrocytic cytoskeleton protein GFAP plays role in many processes in the brain, and they discussed the versatility of the GFAP cytoskeletal network from gene to function with a focus on astrocytes during human brain development, aging and disease^45^. Furthermore, GFAP in Cerebrospinal Fluid (CSF) serves as a potential biomarker of Alexander disease that is comparable between mouse models and human patients^46^.

### Comparison of CD and Deseq2 methods

In order to compare the two methods of CD and Deseq2, we performed differential expression analysis on young and old samples of human Adipose_Subcutaneous tissue using CD and Deseq2 methods respectively. We found overlapping 637 out of the top 2000 DEGs in both CD and Deseq2. That is 32% of the top DEGs were identified using both methods.

## Discussion

In the comparison of age-related genes in multiple tissues of human and mouse, we used GTEx data and more sensitive algorithms than the previous studies, and we found 9 pairs of tissues were significantly correlated between human and mouse on aging. The results were similar to those of Zahn^13^ and Yang^14^.

By functional enrichment analysis of DEGs, we have found some terms related to aging, such as GO:0031012~extracellular matrix^35^, Phosphoprotein^36,37^, Cytoplasm^38^, Cell cycle, Cell division, ATP-binding and GO:0005515~protein binding *et al*.

When we performed a crosswise comparison of 18 tissues in human and mouse respectively, we found that the human Brain_Cortex aging is significantly associated with Brain_Hippocampus aging, which was also found in mouse. Next, we focused on comparing four brain-related tissues in human and mouse, and found a gene–GFAP–related to aging in both human and mouse.

Since human and mouse DEGs are obtained by different algorithms, it is necessary to parallel the two methods over the same dataset to make sense of the impact of technical error. So we applied CD and Deseq2 to analyze the DEGs of human Adipose_Subcutaneous respectively. Also, because we only focused on the overlapping of aging genes in human and mouse, we were not positioned to identify human-specific gene expression changes related to aging. More research is needed to find human specific pathways and mechanisms that contribute longer lifespan in human^47^.

## Materials and Methods

### Data collection

We downloaded human multi-tissue gene expression data from the Genotype-Tissue Expression (GTEx) Portal (https://www.gtexportal.org/home/). And two age-related differential expression gene data from Enrichr (http://amp.pharm.mssm.edu/Enrichr/#stats). These two datasets are Aging_Perturbations_from_GEO_down and Aging_Perturbations_ from_GEO_up which are obtained by applying CD algorithm^48^ to the GEO data (https://www.ncbi.nlm.nih.gov/geo/) to analyze the age-related genes. Comparisons between human and mouse DEGs were based on homologous genes which used HOM_ MouseHumanSequence.rpt obtained from MGI Data and Statistical Reports (http://www.informatics.jax.org/downloads/reports/index.html).

### Matching of tissues

We matched 15 tissues between GTEx data and GEO data, and then compared the DEGs related to aging between human and mouse. In addition, in terms of the GEO data itself, we found three additional human and mouse tissues which are matched. So we collected 15 human tissues from GTEx data, 3 human tissues from GEO data, and 18 mouse tissues corresponding to human tissues from GEO data (see Table 1 and Table 2).

### Data pre-processing

We restricted GTEx RNA-seq tissue-wide expression data to individuals who were 30 or under (young), and 65 or over (old), and removed genes that had either 0 or 1 read in minimal pre-filtering.

### Differential gene expression analysis

We applied Deseq2 to identify age-related genes in humans^24,49^. Deseq2 algorithm has two requirements of inputting data: (1). Deseq2 requires that the input data be a matrix of integers, and (2). the matrix is not standardized. It is worth noting that Deseq2 has its own strategy for calculating the scaling factors. For data visualization purposes, we log transformed our data, and added a pseudo count to avoid undefined values. Deseq2 provides two types of transformation methods for count data: regularized-logarithm transformation (rlog^24^) and variance stabilizing transformation (VST^27^). Both transformations produce transformed data on the log2 scale which has been normalized with respect to library size or other normalization factors^24^. Usually, rlog is used when the data set is less than 30, VST is used for large data sets, and the most appropriate one is automatically selected during the Deseq2 analysis process (Fig. S1). Then, we used the negative binomial distribution to calculate the statistical significance (p-values) among all genes across datasets^50^, and FDR corrected using the Benjamini-Hochberg method^51–53^. Genes were considered differentially expressed if their adjusted p-value < 0.05.

For GEO data, DEGs are obtained by the CD algorithm^48^. In this paper, we directly used the DEGs on GEO data obtained by Wang *et al*.^15^.

### The Fisher’s exact test

For each pair of tissues, the statistical significance of the difference between human aging genes and the mouse aging genes was assessed by fisher’s exact test^54–56^. P-values were corrected for multiple-hypothesis testing using Benjamini-Hochberg correction^51^, with a significance threshold of adjusted p-value < 0.05.

### Gene function enrichment analysis

In this paper, DEGs were annotated by David tools (V6.7) (DAVID; http://david.abcc.cifcrf.gov/)^32,57^ and ClueGO v2.5.1^43^ in Cytoscape^44^. In these two analyses, we adopted the threshold p-value < 0.05.

## Supplementary information

Supplementary information

Dataset 1

Dataset 2

Dataset 3

Dataset 4

Dataset 5
