# Supplementary material for: Comparison of multi-tissue aging between human and mouse
Source: Sci Rep. 2019 Apr 17;9:6220. doi: 10.1038/s41598-019-42485-3 (PMC6470208; doi:10.1038/s41598-019-42485-3)
Supplement: Supplementary file 1 — Supplementary information [file 41598_2019_42485_MOESM1_ESM.docx]

**Supplementary Information**

**Comparison of multi-tissue aging between human and mouse**

Jujuan Zhuang^1,#^, Lijun Zhang^1,#^, Shuang Dai^1^, Lingyu Cui^1^, Cheng Guo^2^, Laura Sloofman^3^, Jialiang Yang^4,^*

^1^School of Science, Dalian Maritime University, Dalian, Liaoning, 116026, P. R. China

^2^Center for Infection and immunity, Columbia University, New York City, New York, United States of America.

^3^Department of Genetics and Genomic Sciences, Icahn School of Medicine at Mount Sinai, New York City, New York, United States of America.

^4^Geneis (Beijing) Co. Ltd., Beijing, 100102, P. R. China.

# The authors contributed equally to this study.

*Correspondence: JialiangYang (Email: yangjl@geneis.cn)

**Supplementary Figure**

**Figure S1:** Count data transformations, for the shifted logarithm log2(n+1) **(a)**, the regularized log transformation **(b)**, and the variance stabilizing transformation **(c)**.


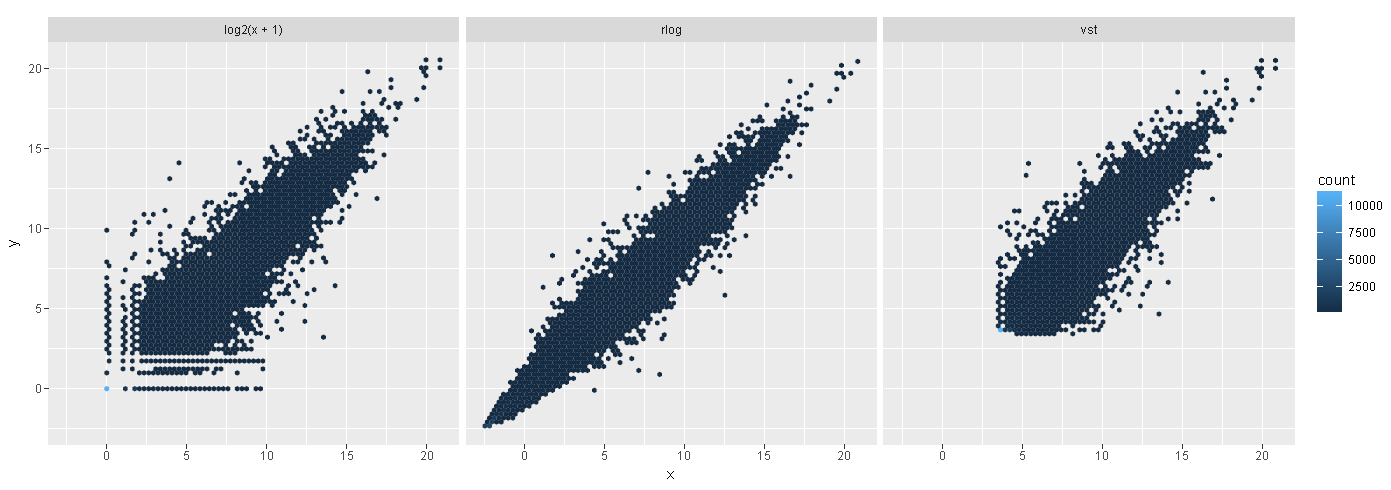


**(c)**

**(a)**

**(b)**

**Supplementary Tables**

**Table S1** Summary of GSE(Series) information for all tissues in GEO database.

| **mouse** | | | | | | |
| --- | --- | --- | --- | --- | --- | --- |
| tissue | search number | | | | | |
| peripheral adipocyte | GSE25905 |  |  |  |  |  |
| bone marrow adipocyte | GSE25905 |  |  |  |  |  |
| thoracicaorta | GDS3077 |  |  |  |  |  |
| cerebellum | GDS4153 |  |  |  |  |  |
| neocortex | GSE13120 | GSE11291 | GDS2973 |  |  |  |
| hippocampus | GSE49699 | GSE48911 | GSE21681 | GSE5078 | GDS3895 | GSE48911 |
|  | GSE14505 | GSE29075 | GDS4019 | GDS2654 | GSE14724 | GSE20270 |
| heart | GSE15129 | GDS3102 | GSE11291 | GSE41018 | GDS2972 |  |
| cardiac ventricle | GSE12480 | GDS399 |  |  |  |  |
| kidney | GSE15129 | GSE41018 |  |  |  |  |
| liver | GSE11097 | GDS1990 | GSE20426 | GSE25325 | GSE3150 | GSE3129 |
|  | GSE20425_ | GSE15129 | GDS2019 |  |  |  |
| Lung | GDS2929 | GSE27964 |  |  |  |  |
| skeletal muscle | GDS2612 | GDS4892 | GSE50821 |  |  |  |
| ovary | GSE14937 |  |  |  |  |  |
| spleen | GSE13148 |  |  |  |  |  |
| Small_Intestine_Terminal_Ileum | GSE39974 |  |  |  |  |  |
| brain | GSE15129 | GSE41018 | GDS1311 | GSE45044 | GSE20411 |  |
| retina | GDS2654 | GSE17423 | GDS2936 | GSE33674 |  |  |
| hematopoietic stem cells | GSE48893 |  |  |  |  |  |
| **human** | | | | | | |
| tissue | search number |  |  |  |  |  |
| brain (frontal cortex) | GSE53890 |  |  |  |  |  |
| hematopoietic stem cells (HSC) | GDS3942 | GSE32719 |  |  |  |  |
| retinal periphery | GSE32614 |  |  |  |  |  |

**Table S2** Overview of human and mouse homologous genes and gene object for fisher's exact test

|  | Total gene | Total homologous gene | Liver |
| --- | --- | --- | --- |
| Human | 56238 | 18090 | 108 |
| Mouse | 45102 | 16039 | 4756 |
| overlap |  | 14212 |  |

**Table S3** The top 10 overlapped terms of aging-related genes in 18 human and mouse tissues obtained by DAVID.

| Heart_Atrial_Appendage | Ovary | | Brain_Cerebellum |
| --- | --- | --- | --- |
| GO:0031012~extracellular matrix | Extracellular matrix | | Alternative splicing |
|  | Phosphoprotein | | Phosphoprotein |
| Spleen | Cytoplasm | | Cell junction |
| GO:0005615~extracellular space | GO:0030017~sarcomere | | Methylation |
|  |  | | GO:0005737~cytoplasm |
| Adipose_Visceral_(Omentum) | Lung | | Heart_Left_Ventricle |
| Phosphoprotein | Phosphoprotein | | Phosphoprotein |
| Acetylation | GO:0005515~protein binding | | GO:0005515~protein binding |
| Ubl conjugation | Acetylation | | Cytoplasm |
| GO:0005515~protein binding | Ubl conjugation | | Acetylation |
| Cytoplasm | Cytoplasm | | GO:0005737~cytoplasm |
| GO:0005737~cytoplasm | GO:0005737~cytoplasm | | Transit peptide |
|  | Isopeptide bond | | Mitochondrion |
|  | Polymorphism | | GO:0005829~cytosol |
|  | GO:0005829~cytosol | | GO:0005739~mitochondrion |
|  | Methylation | | Cell adhesion |
| Liver | Artery_Aorta | | Muscle_Skeletal |
| Cell cycle | Phosphoprotein | | Phosphoprotein |
| Cell division | GO:0005515~protein binding | | Cytoplasm |
| Mitosis | Metal-binding | | Proteasome |
| GO:0051301~cell division | Ubl conjugation | | Acetylation |
| GO:0007067~mitotic nuclear division | Acetylation | | GO:0005515~protein binding |
|  | Cytoplasm | | GO:0005829~cytosol |
| Chromosome | GO:0005788~endoplasmic reticulum lumen | | GO:0005737~cytoplasm |
| Cytoskeleton |  |  | GO:0000502~proteasome complex |
| Centromere | Isopeptide bond | | Nucleotide-binding |
| GO:0005654~nucleoplasm | Cell adhesion | |  |
| GO:0005515~protein binding | Methylation | | ATP-binding |
| Brain_Cortex | Brain_Hippocampus | | brain |
| Phosphoprotein | Phosphoprotein | | GO:0070062~extracellular exosome |
| Cell junction | Alternative splicing | | Membrane |
| Synapse | Cytoplasm | | GO:0043025~neuronal cell body |
| GO:0005829~cytosol | Membrane | | GO:0043209~myelin sheath |
| Cytoplasm | GO:0070062~extracellular | | Synapse |
| GO:0070062~extracellular exosome | exosome | | Phosphoprotein |
|  | GO:0005829~cytosol | | GO:0005615~extracellular space |
| GO:0030054~cell junction | Calmodulin-binding | | GO:0048471~perinuclear region of cytoplasm |
| GO:0005515~protein binding | GO:0005515~protein binding | |  |
| GO:0008021~synaptic vesicle | Cytoskeleton | | GO:0030425~dendrite |
| Transport | GO:0005737~cytoplasm | | GO:0005515~protein binding |
| Adipose_Subcutaneous | | retinal_periphery | |
| Phosphoprotein | | GO:0006413~translational initiation | |
| Extracellular matrix | | GO:0070062~extracellular exosome | |
| GO:0005578~proteinaceous extracellular matrix | | Acetylation | |
| GO:0030198~extracellular matrix organization | | Ribosomal protein | |
| GO:0016020~membrane | | Ribonucleoprotein | |
| Glycoprotein | | GO:0003735~structural constituent of ribosome | |
| Membrane | | GO:0006412~translation | |
| Cell adhesion | | GO:0005840~ribosome | |
| signal peptide | | GO:0006364~rRNA processing | |
| Collagen | | GO:0043209~myelin sheath | |

**Table S4** Number of functions of aging genes in human and mouse tissues obtained by DAVID.

| Human | | Mouse | | Overlap term |
| --- | --- | --- | --- | --- |
| Tissue | Term size | Tissue | Term size |  |
| Adipose Subcutaneous | 118 | peripheral adipocyte | 326 | 30 |
| Adipose_Visceral | 57 | bonemarrow adipocytes | 172 | 7 |
| Artery_Aorta | 107 | thoracicaorta | 415 | 28 |
| Brain_Cerebellum | 56 | cerebellum | 32 | 5 |
| Brain_Cortex | 74 | neocortex | 257 | 40 |
| Brain_Hippocampus | 131 | hippocampus | 515 | 59 |
| Heart_Atrial | 1 | heart | 444 | 1 |
| Heart_Left_Ventricle | 49 | cardiac ventricle | 391 | 21 |
| Kidney_Cortex | 0 | kidney | 442 | 0 |
| Liver | 48 | liver | 610 | 23 |
| Lung | 98 | lung | 377 | 15 |
| Muscle_Skeletal | 95 | skeletal muscle | 302 | 28 |
| Ovary | 43 | Ovary | 233 | 4 |
| Small_Intestine | 0 | Small_Intestine | 139 | 0 |
| Spleen | 6 | Spleen | 37 | 1 |
| brain | 80 | brain | 375 | 49 |
| retinal_periphery | 142 | retinal | 356 | 68 |
| hematopoietic_stem_cells | 5 | hematopoietic_stem_cells | 33 | 0 |

**Table S5** The overlapping homologous aging-related genes among four tissues related to brain in human and mouse

|  | human | mouse |
| --- | --- | --- |
| 1 | GFAP | NOV |
| 2 | CP | BEND6 |
| 3 | CYP2J2 | CYFIP2 |
| 4 | NXPH1 | C4B |
| 5 | NKX2-2 | CLIP3 |
| 6 | ITGB4 | PLIN4 |
| 7 | FGF12 | GFAP |
| 8 | VWF | SEPT5 |
| 9 | MAL2 |  |
| 10 | PCMT1 |  |
| 11 | TTC19 |  |
| 12 | FGF13 |  |
| 13 | JAZF1 |  |
| 14 | RARRES2 |  |
| 15 | KIAA1549L |  |
| 16 | GGT5 |  |
| 17 | FAM102B |  |
| 18 | 4-Mar |  |
| 19 | WSB2 |  |
| 20 | IARS |  |
| 21 | FAM107A |  |
| 22 | BZW2 |  |
| 23 | HPCAL4 |  |
| 24 | PRELP |  |
| 25 | CLDN5 |  |
| 26 | RRAS |  |
| 27 | PRPS1 |  |
| 28 | CAMK4 |  |
| 29 | CALN1 |  |
| 30 | ATP6V1C1 |  |
| 31 | DOK6 |  |
| 32 | RAB3C |  |
| 33 | NEGR1 |  |
| 34 | PCP4L1 |  |
| 35 | XKR4 |  |
| 36 | HHATL |  |
| 37 | SLC9B2 |  |
| 38 | PLXNB1 |  |
| 39 | TOB2 |  |

**Table S6** The overlapping terms among Brain_Cerebellum, Brain_Cortex, Brain_Hippocampus and brain from human donors.

| GOID | Term |
| --- | --- |
| GO:0012505 | endomembrane system |
| GO:0042995 | cell projection |
| GO:0097458 | neuron part |
| GO:0044459 | plasma membrane part |
| GO:0043005 | neuron projection |
| GO:0098590 | plasma membrane region |
| GO:0097708 | intracellular |
| GO:0005737 | cytoplasm |
| GO:0044444 | cytoplasmic part |
| GO:0032879 | regulation of localization |
| GO:0051049 | regulation of transport |
| GO:0006887 | exocytosis |
| GO:0048518 | positive regulation of biological process |
| GO:0023051 | regulation of signaling |
| GO:0050793 | regulation of developmental process |
| GO:0051239 | regulation of multicellular organismal process |
| GO:0007165 | signal transduction |
| GO:0048522 | positive regulation of cellular process |
| GO:0010646 | regulation of cell communication |
| GO:2000026 | regulation of multicellular organismal development |
| GO:0032940 | secretion by cell |
| GO:0046903 | secretion |
| GO:0099503 | secretory vesicle |
| GO:0030030 | cell projection organization |
| GO:0036477 | somatodendritic compartment |
| GO:0120036 | plasma membrane bounded cell projection organization |
| GO:0007399 | nervous system development |
| GO:0022008 | neurogenesis |
| GO:0048699 | generation of neurons |
| GO:0030182 | neuron differentiation |
| GO:0048666 | neuron development |
| GO:0031175 | neuron projection development |
| GO:0006810 | transport |
| GO:0051128 | regulation of cellular component organization |
| GO:0006811 | ion transport |
| GO:0034220 | ion transmembrane transport |
| GO:0005615 | extracellular space |
| GO:0031090 | organelle membrane |
| GO:0043230 | extracellular organelle |
| GO:0031982 | vesicle |
| GO:0098588 | bounding membrane of organelle |
| GO:1903561 | extracellular vesicle |
| GO:0070062 | extracellular exosome |
| GO:0097708 | intracellular vesicle |
| GO:0031410 | cytoplasmic vesicle |
| GO:0044433 | cytoplasmic vesicle part |
| GO:0045055 | regulated exocytosis |
| GO:0007275 | multicellular organism development |
| GO:0009653 | anatomical structure morphogenesis |
| GO:0048731 | system development |
| GO:0031344 | regulation of cell projection organization |
| GO:0060284 | regulation of cell development |
| GO:0007417 | central nervous system development |
| GO:0120035 | regulation of plasma membrane bounded cell projection organization |
| GO:0043168 | anion binding |
| GO:0043209 | myelin sheath |
